# Functional Gels and Chemicals Used in Oil and Gas Drilling Engineering: A Status and Prospective

**DOI:** 10.3390/gels10010047

**Published:** 2024-01-09

**Authors:** Keqing Yang, Yingrui Bai, Jiayun Ma, Jinsheng Sun, Yuan Liu, Youming Lang

**Affiliations:** School of Petroleum Engineering, China University of Petroleum (East China), Qingdao 266580, China; yangkeqing1017@163.com (K.Y.); z23020042@s.upc.edu.cn (J.M.); sunjsdri@cnpc.com.cn (J.S.); kk18252127631@163.com (Y.L.); s22020169@s.upc.edu.cn (Y.L.)

**Keywords:** polymer gel, functional material, formation mechanism, drilling fluid, enhanced recovery

## Abstract

Research into functional gels and chemicals and their applications represents a cutting-edge international field of study. For example, investigating how they can be applied in oil and gas drilling (and extraction engineering) and developing novel functional chemical materials for the oil field could provide innovative solutions and technological methods for oil and gas drilling and extraction operations. Through a literature analysis, this paper presents a review of the current research status and application scenarios of different types of functional gels and chemicals, both domestically and internationally. The classification and preparation principles of various functional materials are systematically outlined and the current applications of functional gels and chemicals in oil and gas drilling and extraction engineering are introduced. These applications include drilling and plugging, enhanced oil recovery, water plugging, and profile control. The formation mechanisms and application scenarios of different types of gels and chemicals are also analyzed and summarized, with a discussion of their prospects in oil and gas drilling and extraction engineering. We broaden the scope of functional gels and chemicals by exploring new application fields and promoting the development of different types of gels and chemicals in a more intelligent direction.

## 1. Introduction

The exploration and development of oil and gas are expanding to deep, ultra-deep, unconventional, and low-grade oil and gas resources. Accordingly, oil exploitation is facing more demanding geological conditions and many technical bottlenecks. For this reason, research on new functional gels and chemicals is key to realizing efficient oil and gas development [1]. Smart materials can respond to changes in their environment in a predetermined way, and research in functional materials is often driven by potential applications [2]. Functional gels and chemicals are of specific technological importance in the face of significant global challenges, such as energy production and storage, sustainability, and health care. It is hoped that their continued development will trigger an epochal and profound materials revolution. Researchers have recently applied smart materials to oil and gas drilling and production engineering fields, in areas such as drilling and plugging, enhanced oil recovery, water plugging, and profile control.

Drilling fluid is a generic term for all types of circulating fluids that meet the needs of drilling work through its multiple functions. With the development of oil and gas resources from conventional to unconventional and ultra-deep oil and gas, drilling fluids are facing increasing technical challenges [3]. Functional drilling fluid flow regulators, filtration loss reducers, and plugging materials can be added to the drilling fluid system according to the formation conditions and engineering needs [4]. Drilling fluids have a high dynamic–plastic ratio and suitable shear dilution, meaning they can cope with environmental changes and can be highly targeted [5]. They also have an outstanding plugging effect and only cause minor damage to the formation [6].

Cementing fluid is used to create a strong and impermeable bond between the casing and the wellbore, ensuring wellbore integrity and preventing fluid migration. Functional chemicals are employed in cementing operations to improve cement slurry properties, enhance zonal isolation, and mitigate potential issues such as gas migration and casing corrosion. Changes in downhole temperature and pressure can alter the filter cake during the cementing of oil and gas wells [7]. Moreover, volume contraction of the cementite can result in the emergence of micro-annular gaps at the diaphragm interface, which can form a pathway for downhole formation fluids to escape. If we suppose that these micro-ring holes cannot be repaired quickly, the micro-fractures continue to extend and develop. Eventually, this can result in the severe consequence of cement sheath plugging failure. Therefore, the development of a new technology to endow the cement sheath with self-diagnosis and self-repair abilities (similar to damage-cementing self-repairing cement technology) has become a hotspot in cementing fluid research in recent years. Such a material would automatically repair micro-fractures and micro-annular gaps in the cement sheath, prevent further oil and gas flow, restore the plugging integrity of the cement sheath, and prolong the production life of the oil and gas wells. Due to their excellent physicochemical properties, nanomaterials were introduced into cement in the 1990s and significantly improved its properties, such as filling the pores of cementitious materials, promoting cement hydration, and improving mechanical strength and durability [8,9].

Fracturing fluid, commonly used in hydraulic fracturing operations, is designed to create and propagate fractures in the reservoir rock, thereby enhancing oil and gas production from unconventional reservoirs. The performance of fracturing fluids predominantly determines the effect of fracturing construction. Clean fracturing fluid is different from guanidinium fracturing fluid by not forming a filter cake due to filtration losses in the formation [10,11]. Moreover, its filtration loss rate does not change over time and it inherits the excellent performance of viscoelastic surfactant fracturing fluids. In addition to causing minimal damage to the formation, viscoelastic surfactant fracturing fluid also has good performance in terms of anti-temperature, anti-shear, sand carrying, glue-breaking return, and filtration loss reduction. Due to their unique physicochemical and surface properties, nanomaterials also exhibit great potential for applications in crosslinking, viscosity enhancement, and the temperature resistance of fracturing fluids, especially in improving the rheology of water-based fracturing fluids [12].

Nano-smart oil drive technology integrates nano and enhanced oil recovery (EOR) technologies. This technology offers advantages that the conventional EOR process does not have, such as a wide wave range, low investment cost, and environmental adaptability [13]. In the fields of high water content, low permeability, tight oil, and shale oil, nanomaterials have a huge potential to improve the recovery of crude oil in complex reservoirs and “difficult to inject, difficult to extract” production problems [14]. Moreover, given that nano oil repellents of small scale and multi-functionality have clear advantages, the question of how they can play a role in different types of reservoirs effectively has become the focus of research.

Profile control involves managing fluid flow within the reservoir to ensure uniform sweep efficiency and maximize oil recovery. With the application of various plugging agents, the production capacity of oilfields has increased over the years. However, due to numerous applications, the range of formations that can be plugged by plugging agents is becoming smaller. The unique response mechanism of smart gels can be utilized to enable them to exhibit the smart features expected from oilfield conditioning [15], such as allowing the fluid to penetrate along the longitudinal direction into low-permeability zone oil formations and increasing the wave volume of the injected water in the low-permeability zone. Ultimately, this will increase the recovery of crude oil in the low-permeability areas of far wells. The main synthetic monomer of the microspheres is polyacrylamide (AM), and this plugging agent is characterized by its ability to expand rapidly and become more extensive in contact with water. After injecting the microspheres into the ground, the plugging effect is evident on the highly porous flow paths or fissure channels.

This paper highlights the use and development of smart gels (responsive polymers), shape memory polymers, nano/micro-sized polymer targets, and application-driven materials. From the perspective of functional chemical materials, the paper also summarizes the classification, mechanism of action, characteristics, and current status of the application of different functional materials in oilfield applications. Furthermore, analyses of R&D directions and the prospect of applications in oilfield development are provided.

## 2. Functional Drilling Fluid Chemicals

Drilling fluids generally contain a water-based drilling fluid (WBDF), an oil-based drilling fluid (OBDF), and a synthetic-based drilling fluid (SDF). The use of oil- and synthetic-based drilling fluids is often restricted due to environmental pollution concerns and high costs. In contrast, water-based drilling fluids are becoming the preferred solution due to their advantages of low cost, environmental friendliness, and easy biodegradability [5]. However, the performance of water-based drilling fluids can be reduced due to the significant increases in temperature and pressure caused by deep ultra-deep drilling. The performance of the treating agent largely determines the performance of the drilling fluid [4]. When using traditional water-based drilling fluid treating agents in high-temperature formations (or drilling in saline formations), the efficiency of the work is greatly reduced and the performance may even fail, resulting in frequent accidents that can affect normal drilling [16]. Therefore, it is very important to add a treating agent with different functionality to the drilling fluid system [17].

### 2.1. Drilling Fluid Flow Regulator

Drilling fluid flow regulators are treatment agents used to improve the rheological properties of drilling fluids by increasing the viscosity and lifting cuttings, ensuring the drilling fluid has a higher dynamic–plastic ratio and better shear dilution. However, with the development of large displacement wells, large inclination directional wells, and deep/ultra-deepwater wells (and other technologies), the rheology of drilling fluids in complex formations is still prone to problems with viscosity, insufficient cutting force, rapid performance decay, or a loss of suspension and carrying capacity [18]. These issues result in insufficient cleaning of the borehole in huge displacement and complex wells. One of the biggest problems facing deep/ultra-deepwater drilling is that drilling fluids have to operate over a temperature range of 4–150 °C, causing drastic changes in the rheological properties [2]. Weakening the sensitivity of drilling fluid viscosity and shear force to temperature and maintaining the rheological parameters within a specific temperature range are keys to solving this problem [19].

A synthetic-based mud (SBM) with constant rheological properties was first prepared by Van Oort et al. [20]. This novel material displays a continuous (“flat”) rheological profile over a broad temperature and pressure range, unlike conventional SBMs. Flat rheological behavior refers to a material’s response to shear stress where the viscosity remains constant over a wide range of shear rates [21]. For instance, when the temperature and pressure are adjusted, several important rheological parameters essentially remain the same, including the 6 rpm reading, yield point, and gel strength. Moreover, due to the flat rheology, a higher viscosity can be maintained without having an adverse impact on the drilling rate or equivalent circulating density (ECD). Additionally, the barite suspension characteristics and cutting carrying capacity are substantially enhanced. Young et al. [22] used a new emulsifier and rheology modifier package to overcome some of the drawbacks of the first-generation synthetic-based drilling fluid system developed by Van Oort et al. [20], such as poor temperature resistance. The emulsifier has a new chemistry to deliver emulsification and surface-wetting capabilities while improving the solid tolerance. This new rheology modifier has a different chemistry, is more effective and thermally stable, and imparts less impact on gel strengths compared to previous rheology modifiers. The improved second-generation system also has a simpler formulation and a lower gel strength, with a temperature resistance of up to 176.7 °C.

One method of preparing a smart drilling fluid flow regulator is to introduce temperature- or salt-sensitive polymer monomers into synthetic polymer-based rheology modifiers and use their spontaneous behavior to regulate the rheology of drilling fluids at different temperatures or mineralization conditions [23,24].

**Figure 1:**
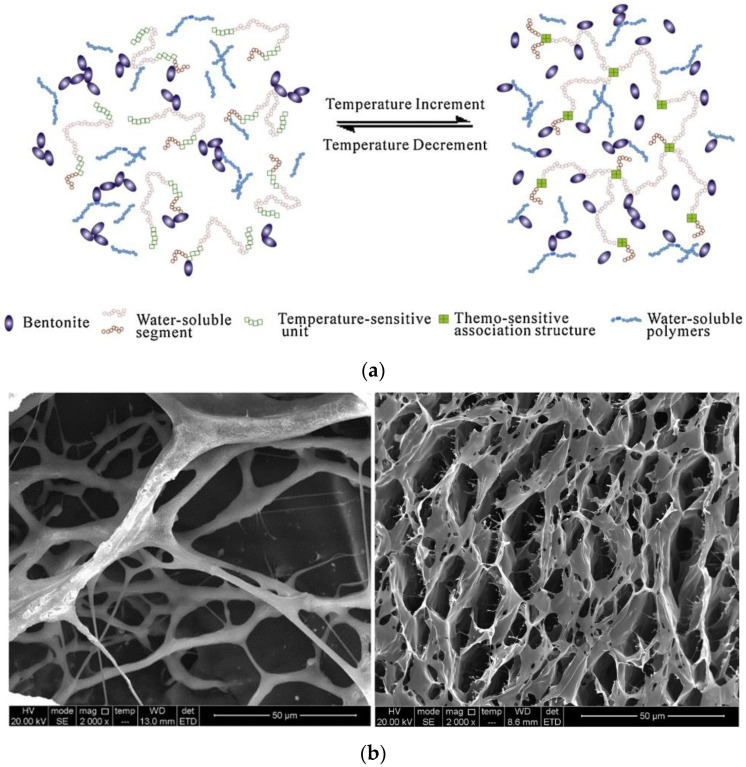
(**a**) Rheological control process of thermo-sensitive polymer for drilling fluid at different temperatures. The morphology of 0.8%P ANA in aqueous solution at different temperatures through ESEM (**b**) at 25 °C and 60 °C [27].

Temperature-sensitive polymers can be effectively used in water treatment and as flow modifiers in drilling fluids to achieve constant rheology due to their significant hydrodynamic volume and molecular conformation changes in response to temperature [25,26]. Xie et al. [27] synthesized a novel temperature-sensitive copolymer (PANA) of acrylamide (AM), sodium 2-acrylamido-2-methylpropane sulfonate (NaAMPS), and N-vinyl caprolactam (NVCL), which was synthesized by free radical polymerization under optimal conditions. Temperature has an opposite impact on the viscosity of polymer and bentonite particles, as displayed in Figure 1a. Therefore, the synergistic interaction of the polymer and bentonite particles could produce fluids with a “flat” characteristic. Changes in the molecular chain due to temperature were intuitively examined by FEI ESEM Quanta 450 to understand the mechanism by which PANA controlled the rheological behavior of fluids. The micro-morphology of a (0.8%) polymer aqueous solution at 25 and 60 °C is displayed in Figure 1b, where it is evident that a continuous network structure developed in the sample. This indicated that PANA has some ability to modify the rheology of the fluid. The molecular chain’s skeleton became much more prominent as the temperature increased from 25 to 60 °C, and the network structure became significantly denser. As a result, the thermal viscosity of the polymer solution increased from 1403 to 2482 mPa-s.

When drilling in highly saline environments (such as salt paste formations), the occurrence of the “polyelectrolyte effect” can easily cause conventional polymer treatments to fail, resulting in the destabilization of the drilling fluid system [28]. Moreover, the addition of salt-sensitive polymers can (to a certain extent) increase the ability of drilling fluids to resist salinization. A salt-responsive zwitterionic polymer (PAMN) was developed by Sun et al. [29]. The authors conducted free radical copolymerization in an aqueous solution with [2-(methacrylate)ethyl] dimethyl-(3-sulfopropyl) ammonium hydroxide (MEDS, 97%), N-Vinyl-ε-caprolactam (NVCL, 99%), and acrylamide (AM, 99.8%). Due to the charge interaction on PAMN’s molecular chain being shielded by sodium chloride electrolyte, which increased the chain’s radius of gyration (Rg), the saturated brine drilling fluid’s apparent viscosity and plastic viscosity increased by 542.9% and 925%, respectively, compared to saturated brine drilling fluid. This effectively reduced the viscosity loss of drilling fluids in high-mineralization environments.

### 2.2. Filter Loss Reducer for Drilling Fluids

Drilling fluids suffer from filtration losses, which often cause the clay in the formation to swell and the wall of the well to destabilize, increasing the operation cycle and cost. Accordingly, filter loss reducers are commonly used chemical treatments in drilling fluids to reduce the amount of filtration losses. Reducing filtration losses of the drilling fluid to the formation is an important task in drilling engineering. With the increasing number of deep and ultra-deep wells, the high-temperature environment at the bottom of these wells deteriorates the performance of filter loss reducers or even renders them ineffective. Hence, the stability of filter loss reducers under high-temperature conditions has attracted significant research attention.

Thaemlitz et al. [30] synthesized a terpolymer filter loss reducer through aqueous solution polymerization, with N-vinyl caprolactam, vinyl sulfonic acid, and AMPS as the preferred reactive monomers. In addition, they developed a new environmentally friendly high-temperature-resistant water-based polymer drilling fluid system by using this as the primary agent. In the evaluation experiment at 230 °C for 16 h, the filter loss reducer effectively maintained the rheological stability of the drilling fluid and had a good filter loss reduction effect that could be applied to drilling fluid systems such as seawater and lime mud.

**Figure 2:**
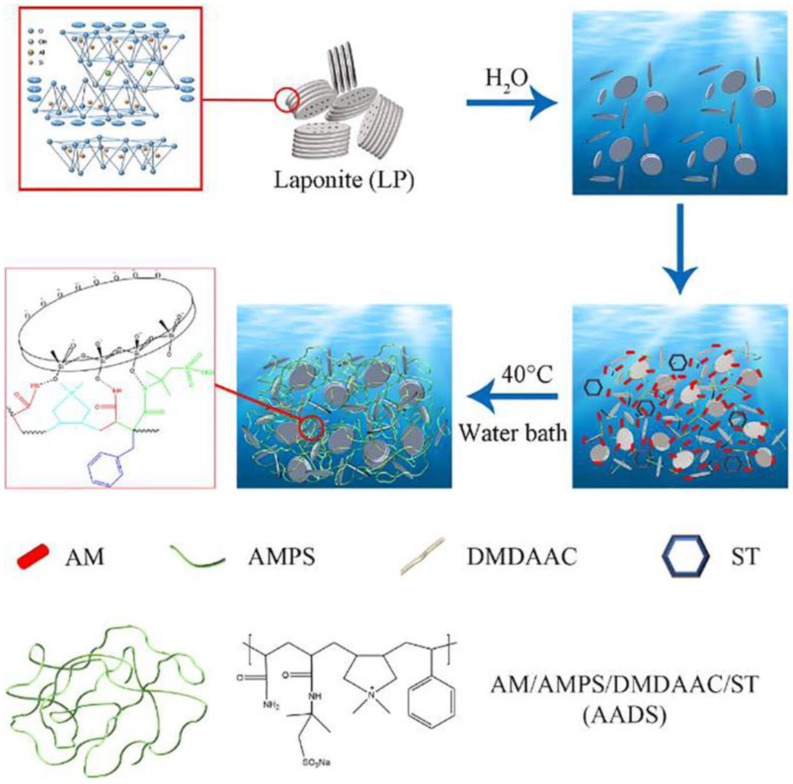
Schematic representation of fabrication of AADS@LP composite [31].

**Figure 3:**
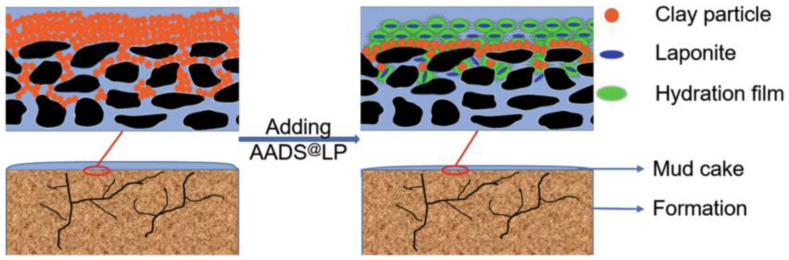
Schematics of the working mechanism of AADS@LP in WDFs [31].

Some of the existing filter loss reducers can meet the needs of the field and effectively reduce filtration losses under high-temperature conditions when contaminated by calcium salts. However, the filter loss reduction effect of drilling fluids deteriorates significantly under these conditions [22], meaning it is necessary to use these reducers in combination with other anti-salt filter loss reducers. Therefore, there is an urgent need to develop a high-temperature and calcium salt-resistant functional drilling fluid filter loss reducer. A nano-lamellar silicate composite (AADS@LP) was prepared by Shen et al. [31]. through in situ emulsion polymerization using acrylamide, 2-AM-2-methyl propane sulfonic acid, diallyl dimethyl ammonium chloride, styrene, and a synthesized chemically pure lithium-montmorillonite clay. A schematic diagram of the preparation is depicted in Figure 2. The AADS@LP acted as a plugging agent for the pore spaces and fractures and could be adsorbed on the surface of clay particles, forming a hydration film as a barrier to prevent clay particle aggregation. This increased the temperature point of clay particle aggregation to 210 °C and improved the stability of clay particles under high-temperature and high-salinity conditions. In the high-temperature and high-polarity solution, the network strength of the polymer was improved, and the sulfonic acid group forced the clay particles to pile up tightly and block the pores and fractures, forming an isolation membrane that effectively reduced the filtration losses. The working mechanism of this process is schematically demonstrated in Figure 3.

Through copolymerization of vinyl amide (VA) and vinyl sulfonate (VS) monomers, Tomislav et al. [32] obtained Hostadrill 4706, which is a new generation of high-temperature-resistant filter loss reducers with temperature resistance exceeding 230 °C. In-house experiments demonstrated that this filter loss reducer had a relative molecular mass ranging from 5 × 105 to 10 × 105 and excellent anti-salt properties, and it could significantly improve the rheological properties of drilling fluids.

### 2.3. Drilling Fluid Plugging Materials

Drilling fluid loss is a phenomenon where drilling fluids are lost in the drilled formation in large quantities during drilling construction, and it is a common drilling engineering malignant accident. Currently, plugging materials used in the field have not fundamentally solved the problems of the poor adaptability of various types of plugging materials in complex formations and their weak ability to stay in the losing channel. Thus, loss accidents have not been effectively controlled and eliminated. Smart drilling fluid plugging materials have become a new research direction in oil extraction. In recent years, researchers have applied smart materials to the field of drilling fluid plugging and developed smart shape memory materials, smart gel materials, and smart bionic materials to further improve performance in terms of loss prevention and plugging. Unlike traditional plugging materials that directly bridge the plugging, the special feature of smart plugging materials is that they can cope with environmental changes. Moreover, they have the characteristics of strong targeting, an outstanding plugging effect, and low damage to the formation [33,34].

Current research on smart plugging materials is mainly categorized into smart gels and shape memory materials. Of these, smart gels can respond differently to various external stimulus sources (commonly temperature [15], pH [35,36], and electric field [37,38] chemical reagents) or condition changes [39]. Smart gels also have relatively good mobility, are unaffected by the pore size of the formation, and have strong adaptive ability.

**Figure 4:**
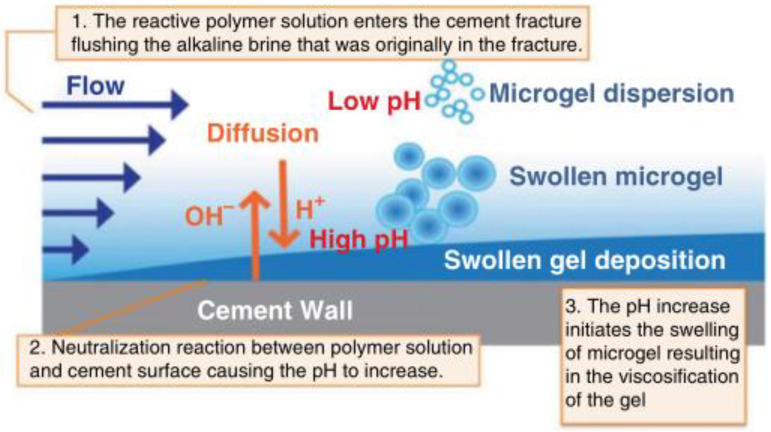
An illustration of the reaction between the pH-triggered microgel dispersion and the cement fracture [40].

To address issues such as the potential loss of carbon dioxide through cement-fractured wells in subsurface formations, an aqueous dispersion of an insoluble smart gel was developed by Ho et al. [40]. This was prepared from pH-sensitive polyacrylic acid polymers and could be injected into cement fractures at low pH, such as water. The pH and volume of the microgel dispersion increased, while OH-ions leached from the alkaline cement walls, neutralizing the solution and causing the gel to become viscous. The swollen gel was subsequently deposited in the cement fractures and exhibited semi-solid properties, which could be used to seal losing channels in cement fractures. The reaction schematic is displayed in Figure 4. From the experiments, the authors concluded that the swollen microgel was transparent and its viscosity increased after neutralization with the cement, which allowed the formation of a highly swollen gel with a large yield stress. Moreover, the pressure gradient could be maintained at an average of 70 psi/ft, preventing fluid flow. Accordingly, it could effectively seal the losing channel under high-pH conditions.

**Figure 5:**
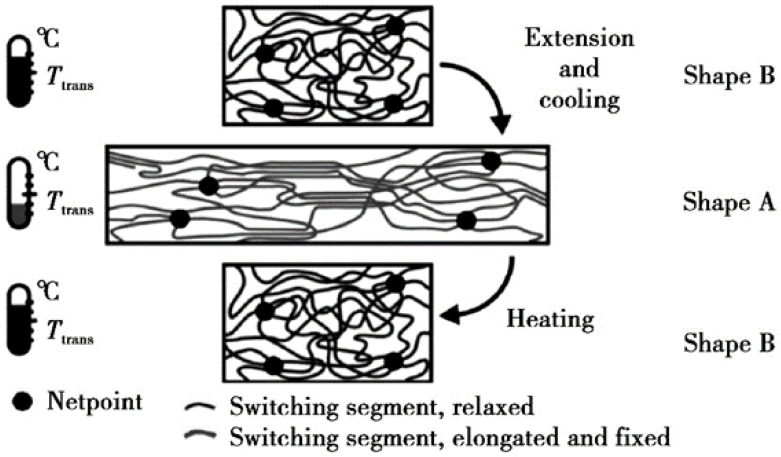
Schematic diagram of the temperature-sensitive deformation mechanism of shape memory polymers [43].

Compared with smart gels, there have been more studies on shape memory materials. Shape memory polymer plugging materials have the advantages of large deformation volumes and low raw material costs. In addition, they can protect the reservoir from contamination and can effectively address the loss problem of complex structural formations [41]. Smart plugging working fluids based on shape memory polymer (SMP) materials are automatically activated by temperature stimulation and produce deformation changes when they enter formations. Accordingly, they can efficiently seal formation pores and solve drilling fluid loss problems without destroying the formation [42]. Andreas et al. [43] explored the mechanism of the action of SMP in more depth in terms of chemical structure. The thermally driven SMP was analyzed with regard to the specific compositional structure of the polymer. Figure 5 displays a schematic diagram of the mechanism and process of thermally induced SMP shape memory.

**Figure 6:**
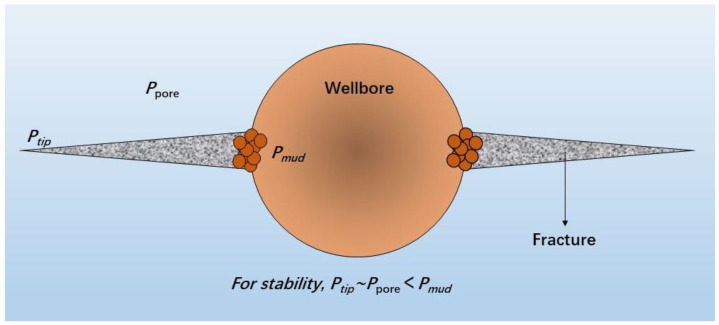
Smart LCM sealing fracture mouth and providing compressional forces to strengthen the wellbore.

To manage the expanding force and functioning of injected lost circulation material (LCM) remotely, Mansour et al. [44] proposed a novel type of smart expandable LCM. This smart LCM comprised anionic shape memory polymers and was activated when natural heat was produced, allowing it to seal a fracture’s width efficiently (without harming production zones) and reinforce the wellbore. As displayed in Figure 6, the smart LCM is activated by the temperature of the bottom hole and can successfully seal the fracture’s mouth or, in some situations, its tip. Therefore, it can effectively seal the fracture width without damaging the production layer, reinforce the borehole by increasing the circumferential stress of the borehole, and successfully seal wedge-shaped slit plates with a slit width of 1.00 to 2.54 mm and a bearing pressure of 34 MPa [45,46]. Table 1 shows the statistics of the application status of functional drilling fluid chemicals. The function characteristics and applications of different functional materials are introduced in detail, which provides a solid theoretical basis for the wide application of drilling fluid.

## 3. Functional Cementing Fluids

Cementing is a crucial step in the construction of oil and gas wells. This process involves running a casing of a specific size into the wellbore and, upon reaching the end of the casing, injecting cement slurry into the annular space between the casing and the wellbore wall to form a cement sheath. A high-quality cement sheath should maintain long-term plugging integrity, which is paramount for the production life of oil and gas wells and directly affects subsequent hydrocarbon extraction. The quality of cementing is directly related to the lifespan and safety of oil and gas fields and wells. Moreover, cementing technology should address the requirements of complex geological conditions, challenging operational environments, and the long-term plugging of cement sheaths. Therefore, further research into smart cementing materials is essential.

### 3.1. Self-Healing Cementing Material

Self-healing materials have a long history of research and application in the field of materials science and engineering [47]. These materials use the principle of bionics that imitates animals healing their injuries after tissue injury to solve the problem of internal microscopic damage in materials. By applying self-healing materials to cement paste, a self-healing cement paste with self-healing properties can be designed. This self-healing cement paste can automatically repair micro-fractures and micro-ring gaps in the cement sheath without interrupting production, prevent further flow of oil and gas, restore the plugging integrity of the cement sheath, and prolong the production life of oil and gas wells.

The secondary hydration of cement stone only has a self-healing effect if the fracture gap is very small, and usually needs to be combined with fiber such as the relatively low engineering cost of the physical external filler synergistic use. Nguyen et al. [48] investigated the self-healing properties of cementite doped with alkali slag-based polyethylene fiber. It was revealed that the fracture recovery rate of this material was higher than that of alkali-activated slag-based cementite. The composite swelling mineral PC90M5B5 was added to a cement paste system by Qureshi et al. [8] and its effect on the self-healing properties of cementite was investigated. After acting for 28 d under the condition of using a high-water–cement-ratio slurry preparation, the strength recovery rate and fracture plugging efficiency of the cement mixes containing swelling minerals increased by 60% and 95%, respectively, demonstrating better self-healing effects.

**Figure 7:**
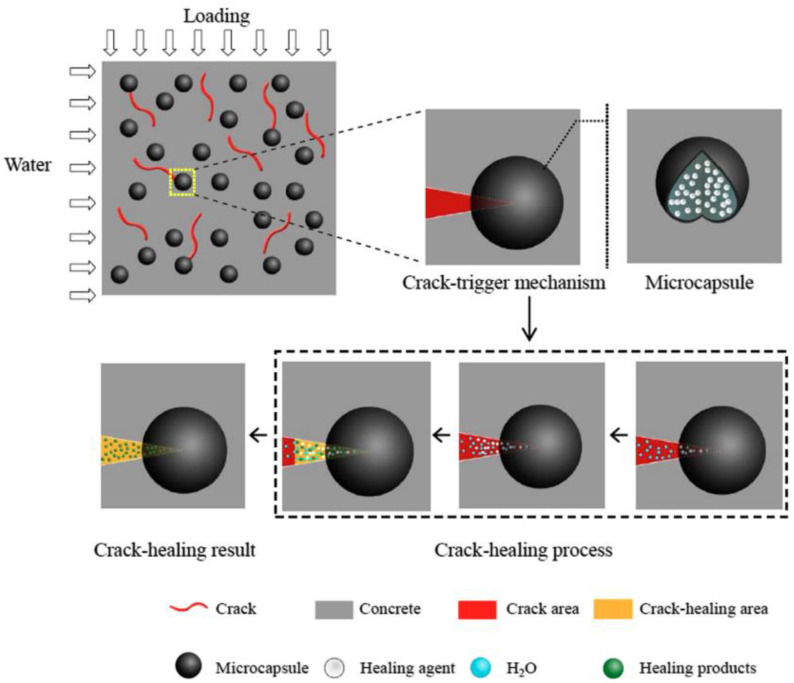
Schematic illustration of the crack-healing process [49].

In oilfield cementing operations, the microfractures created in the cement sheath cannot be expanded due to space constraints. Here, the fractures can often be filled by mixing certain oil- and water-expanding materials with the cement during the slurry preparation process. Fang et al. [49] used calcium sulfate cement as a matrix and selected the self-healing agent CSA (water-absorbent gel) capsule encapsulation for a self-healing test of cement stone. The fill of the hydration product produced by the CSA and water dramatically repaired the first fractures. However, the movement of water from the surface to the body of the cementitious materials significantly affected the amount and speed of the self-healing process. According to the volume fluctuation of the fractures, the healing effectiveness could reach 82.60%. Figure 7 depicts the fracture self-healing procedure.

**Figure 8:**
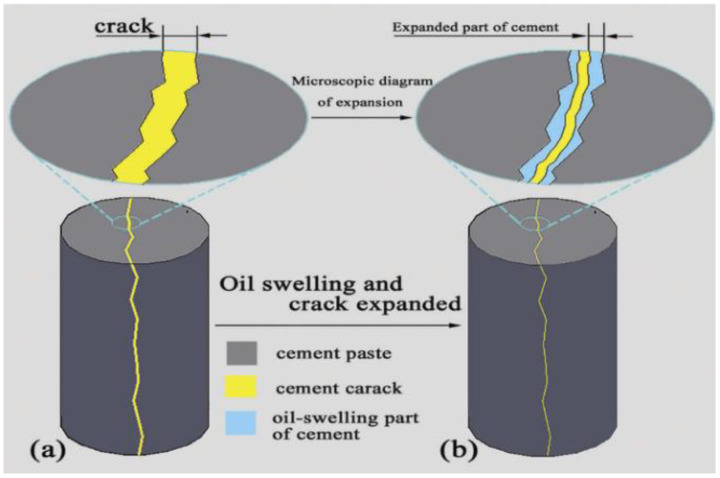
Self-healing mechanism of self-healing cement paste (SHCP). (**a**) means SHCP before oil swelling; (**b**) means SHCP after oil swelling [50].

A new additive oil swelling material (OSM) for self-healing cement paste in oil wells was synthesized by Zhang et al. [50] and was used to evaluate the effect of self-healing triggered by the oil phase during fracture formation. Due to the oil-absorbing swelling of OSM (resulting in a squeezing effect), the self-healing cement paste expands. Then, the expansion partially occupies the space of the fracture, gradually reducing the size of the fracture. The space can even be completely occupied, achieving the effect of repairing the fracture. This self-healing mechanism is presented in Figure 8. Through a test using OMS as a self-healing agent, the cement paste achieved a compressive strength recovery rate of 70.5%, achieving excellent self-healing performance and strong applicability.

### 3.2. Nanoparticle Cementing Material

In recent years, the application of nanomaterials in cementing engineering has developed rapidly. Currently, nanomaterials are mainly used to improve the physical and mechanical properties of cementing materials through particle size grading and bonding. This improves the overall performance of cementing materials by taking advantage of the small particle size, high surface activity, many surface defects, and strong chemical activity.

The physical properties of cement paste (especially strength and permeability) depend largely on its pore structure. Ultrafine particles of nano-SiO_2_ can fill the voids of its structure and result in a more uniform distribution of hydration products [51], improving the durability and strength of cement composites [52,53,54]. Sadrmomtazi et al. [55] studied the mechanical properties, rheological properties, durability, and microstructure of cement paste systems containing additives such as nano-SiO_2_ and silica fume. The results indicated that the addition of nano-SiO_2_ to the system significantly improved the stability of the cement paste, the compressive strength of the cement stone, the splitting, tensile, and flexural strengths, and microstructure densification. In addition, Valipour et al. [56] focused on the effect of nano-SiO_2_ on the permeability of cement stone. Nano-SiO_2_ has a higher surface activity to promote the development of early strength in cement stone. Moreover, nano-SiO_2_ can significantly improve the stability of cement paste, including its compressive, splitting, tensile, and flexural strengths, as well as microstructural density, particularly enhancing the early strength of cement stone. Moreover, there are improvements to the mechanical properties of cement stone compared to silica flour cement paste systems. Finally, nano-SiO_2_ can significantly reduce the porosity of cement stone by blocking the inter-particle pores through particle grading and participating in the hydration reaction of cement. This improves the microstructure of cement stone, rendering it denser with a more uniform pore distribution.

**Figure 9:**
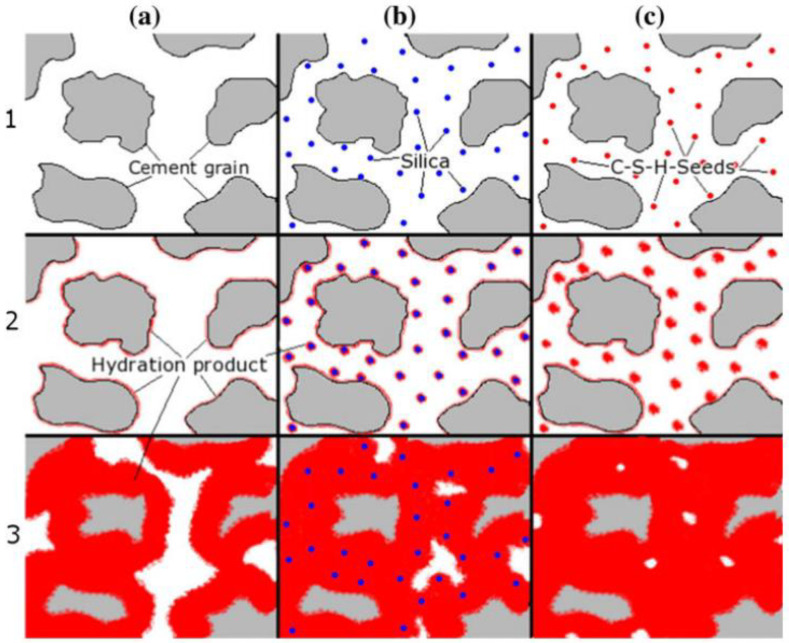
Hydration process of pure cement (**a**) and with the addition of nano-silica (**b**) and C–S–H-seeds (**c**) at different times (1–3) after mixing [57].

Land and Stephan [57] investigated the mechanism of nano-SiO_2_ to promote cement hydration and enhancement using FTIR, TG-DTG, SEM, isothermal calorimetry, and in situ XRD. This was achieved by comparing the hydration process of pure cement with the addition of nano-silica and c-s-h seeds at different times. Figure 9 presents a comparison of the hydration of cement grains with and without additives (a) and the hydration after adding silica or C-S-H particles (b, c). The acceleration could be improved by adding C-S-H particles, which act as direct nucleation seeds, because of the time required for nucleation processes. It is believed that the nanoscale and large specific surface area of nano-SiO_2_ enable it to act as a nucleating agent in the system and promote the hydration of C_3_S and C_2_S to generate more C-S-H phases. Moreover, the tiny size of nano-SiO_2_ can fill the micropores of a C-S-H structure and make the cementite structure denser. Therefore, the filling effect of nano-silica (the volcanic ash effect) and the promotion of the development of crystal nuclei can significantly improve the hydration of cement, improve the microstructure of cement stone, and promote the development of strength in the cement stone, especially the rapid development of early strength. The statistics of the application status of functional cementing materials are shown in Table 2. The functional characteristics of self-healing cement and nano-cement are introduced in detail.

## 4. Functional Hydraulic Fracturing Fluids

Hydraulic fracturing fluids are used in fracturing operations for low-permeability formations to increase the permeability of the formation. Conventional fracturing fluids mainly use a gel system formed by crosslinking natural guanidinium with transition metals as the thickener. When the formation fractures and the proppant enters the action position, the gel system is degraded by injecting oxidizing agents or enzyme accelerators and then returned to the formation. With the increasing demand to improve the fracturing effect, the fracturing fluid system is gradually developing toward multi-functionalization.

### 4.1. Clean Hydraulic Fracturing Fluids

Clean hydraulic fracturing fluids, also known as viscoelastic surfactant fracturing fluids (VES fracturing fluids), perform exceptionally well during the extraction of oil and gas due to several advantages. These include the thickening agent’s simple molecular structure, good solubility, minimal formation damage, and good gel-breaking properties. Currently, clean fracturing fluids are widely employed in low- to medium-temperature formations and have achieved good production increase results. However, there are fewer reports of them being applied in medium- and high-temperature formations above 80 °C [10,11].

**Figure 10:**
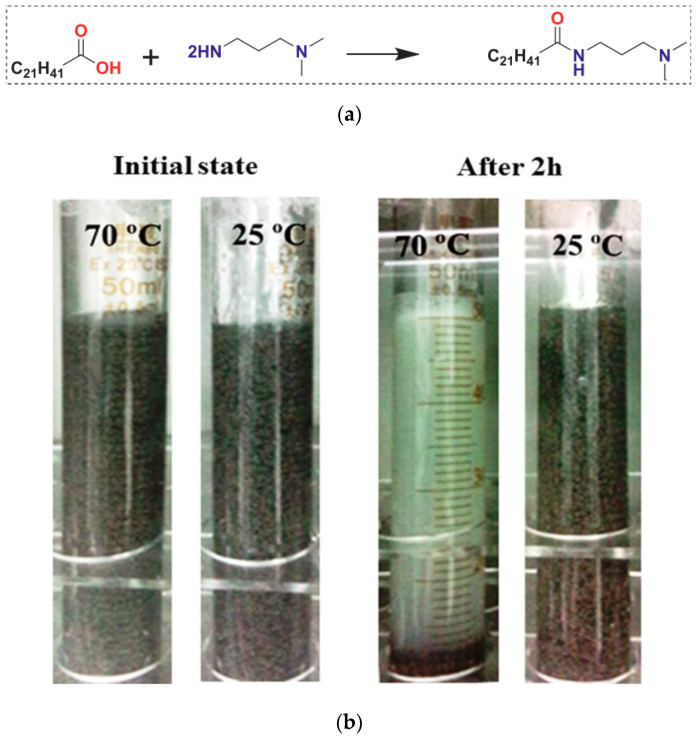
(**a**) Synthetic route of EA surfactant. (**b**) Proppant suspension experiment of 3% EA + 0.6% Nas fracturing fluid at different temperatures [58].

CO_2_-responsive VES schemes offer superior advantages with good CO_2_ responsiveness and switchable system viscoelasticity, signifying the reusability of the fluid. A CO_2_-responsive surfactant isopropyl dimethylamine (EA) was synthesized by Wu et al. [58] using the synthetic route presented in Figure 10a. This was used as a thickener to develop a new viscoelastic surfactant-based fracturing fluid. The viscoelastic properties of this CO_2_-responsive VES fracturing fluid were excellent, which is essential for proppant movement. By adjusting the amount of CO_2_, switching the fluids from high (up to thousands of mPa) to low (less than 3 mPa) was simple. The fluids exhibited good salinity tolerance since the addition of inorganic salt ions only slightly reduced the zero-shear viscosity. Within the measured temperature range (25 to 70 °C), this VES fluid was similarly responsive to CO_2_ pressure. Under supercritical CO_2_ circumstances at 70 °C, the viscosity of the VES fluid was denser than under CO_2_ gas conditions. The gel structure in this VES fluid fully disintegrated in 1 h when the temperature increased to over 70 °C at ambient CO_2_ pressure. Low core damage demonstrated the formation and fracture-friendliness of this VES fracturing fluid.

**Figure 11:**
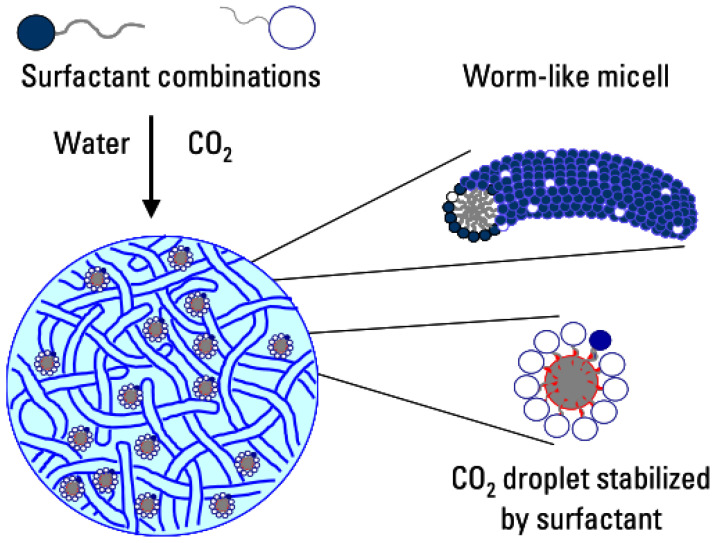
Illustrative diagram of VES fluid–supercritical CO_2_ emulsion stabilization [59].

A supercritical CO_2_-clean foam fracturing fluid was developed by Chen et al. [59] to combine the advantages of VES fluids and CO_2_ emulsification systems. This system combines the advantages of clean fracturing fluids and CO_2_ foam fracturing fluids and can be extended to water-sensitive formations, preventing potential hydro locking. When CO_2_ enters the solution, the surfactant group in the system migrates to the bonding point with the aqueous phase to stabilize the CO_2_ in the aqueous phase (Figure 11). This prevents it from affecting the internal structure of the micelles and altering the viscoelasticity of the fluids, effectively maintaining the stability of the system.

### 4.2. Nanoscale Hydraulic Fracturing Fluids

When developing fracturing fluids that are applicable in tight reservoirs, the question of how to improve the performance of the fracturing fluids and the study of functional fracturing fluids have become important research directions in this field. Nanomaterials have unique physical and chemical properties (such as size effect, strong adsorption, and surface modifiability), allowing them to penetrate deep into the reservoir without being constrained by the pore throat size. In addition, through the design and modification of nanoparticles, they can be made to have certain positive effects on the original surface interface properties of tight reservoirs and residual oil. Therefore, they have been widely employed in water-based fracturing fluids in recent years [60].

Nanoparticles were proposed to be introduced into VES by T. Huang [61] and J.B. Crews et al. [62] to reduce filtration loss and improve temperature resistance, negating the limitation that clean fracturing fluids are mainly applied in medium- and low-temperature formations. Gurluk et al. [63] added MgO and ZnO nanoparticles into VES clean fracturing fluids to obtain nano-modified clean fracturing fluids. The addition of nanoparticles improved the thermal stability of the VES micelle structure up to 135 °C in CaBr_2_ and CaCl_2_ brines and exhibited higher viscosity yield at different shear rates. Moreover, the addition of nanoparticles improved the temperature resistance of the fracturing fluid.

**Figure 12:**
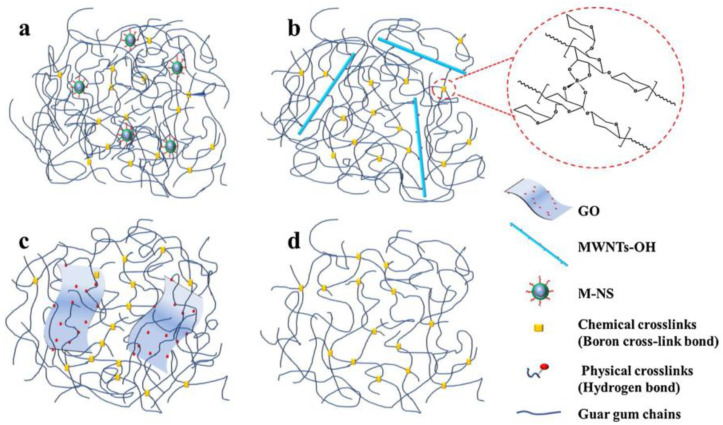
Network structures of M-NS (**a**), MWNTs-OH (**b**), GO (**c**), and blank (**d**) fracturing fluid [65].

Adding nanomaterials to polymer solutions or crosslinked freeze gel fracturing fluids can enhance the spatial network structure of the system and improve the rheology and stability of the fracturing fluid under high temperatures and pressures. Therefore, adding nanomaterials can effectively reduce the amount of polymer, reducing the amount of gel-breaking residue and decreasing the amount of damage to the reservoir and fracture conductivity. Fakoya and Shah [64] revealed that incorporating SiO_2_ nanoparticles into a guar gum solution increased the apparent viscosity and viscoelasticity of the solution due to the physical adsorption of guar molecular chains on the surface of the SiO_2_ nanoparticles, forming a three-dimensional network structure of the system. Different morphology nanoparticles (e.g., SiO_2_ nanoparticles, multi-walled carbon nanotubes, and graphene) were incorporated into guar jelly fracturing fluids by Liu et al. [65] and the effect on their properties was investigated. The nanomaterials acted as crosslinking agents in the hydrogel and interacted with the polymer chains of the hydrogel to form hydrogen and ionic bonds. The experimental results suggested that SiO_2_ nanoparticles had the most significant enhancement effect on the performance of the fracturing fluid system. This manifested as optimizing the shear and temperature stability (120 °C) and increasing the viscosity of the fracturing fluid by nearly 50%. A comparison of the network structure is displayed in Figure 12. Here, the SiO_2_ nanoparticles acted as a nucleation point and a backbone in the fracturing fluid system, resulting in improved fracturing fluid performance.

### 4.3. High-Temperature-Resistant Hydraulic Fracturing Fluids

Reservoir reforming is the core “tool” for high-temperature and deep-seated exploration and development. The questions of how to increase the construction displacement, reduce the fluid friction, improve the sand-carrying capacity, and adapt to the ultra-high-temperature fluids above 200 °C are key challenges for effective reservoir reforming, construction efficiency, and cost reduction of fracturing and acidizing. Here, high-temperature clean fracturing fluid with a resistance up to 240 °C is the “necklace” technology of the fluid material for reservoir reforming. Among these factors, high-temperature resistance and clean fracturing fluid above 240 °C is the “neck” technology of reservoir reforming. High-temperature-resistant hydraulic fracturing fluids are mainly composed of additives, such as high-temperature-resistant thickeners, high-temperature-resistant crosslinking agents, and temperature-stabilizing additives [66].

Thickener systems are generally natural or synthetic polymers that provide the fracturing fluid base fluid with the necessary rheological viscoelasticity of the system structure to achieve sand suspension [67]. After chemical crosslinking, polyacrylamide (HPAM) molecules form a frozen gel with improved viscoelasticity and gel-breaking properties and less residue. The efforts to produce modifications that improve the temperature resistance of polyacrylamide-based polymers have mainly focused on chemical reactions to design poly-copolymers containing branched polymerization monomers or sulfonic acid groups. The aim is to overcome the effects of factors such as salt content and pH on conventional and partially hydrolyzed polyacrylamides. A temperature-resistant thickener was synthesized by Funkhouser et al. [68] using acrylic acid, acrylamide, and 2-acrylamido-2-methylpropanesulfonic acid. Ultimately, the authors developed a fracking fluid system with an extremely excellent temperature resistance of 232 °C.

The addition of a crosslinking agent can convert the original polymer with a linear structure in the fracturing fluid into a type of polymer hydro-frosting material in which linear and reticular structures coexist [69]. Liang et al. [70] proposed a new type of high-temperature-resistant, water-based, crosslinking polymer fracturing fluid with a thickening agent polymerized with AA, AM, and AMPS and a crosslinking agent with organic zirconium as the crosslinking agent. Crosslinking polymer fracturing fluid has a high degree of viscosity retention after 40 s-1 shearing for 80 min at high temperatures of 148–204 °C. Furthermore, the maximum operating temperature of this fracturing fluid was 232 °C.

The addition of temperature-stabilizing additives can significantly improve the temperature resistance of fracturing fluid systems through the mechanism of oxygen depletion (or enhancement) of the spatial network structure of the system. A new high-temperature fracturing fluid stabilizer (phenothiazine) was prepared by Carman and Gupta [71] and applied as a temperature stabilizer compounded with sodium thiosulfate in a specific polymer fracturing fluid system that could withstand formation temperatures of up to 260 °C. The polymer thickener was co-polymerized with acrylamide or its derivatives, vinyl phosphate, and AMPS or other acryloyl amino sulfonates. Moreover, a complex crosslinking agent of organic zirconium–titanium–aluminum was employed. Table 3 summarizes the functional materials in different fracturing fluids.

## 5. Functional Oil Displacement Agents

Nano-smart oil repellent technology integrates nanotechnology and EOR technology and has advantages that the conventional EOR process does not possess, such as wide reach, low investment cost, and strong environmental adaptability. The design ideas employed are as follows: using the “micro-scale” characteristics of the NPs’ oil repellency to achieve the purpose of wide reach; using the “strong hydrophobic and oleophilic” characteristics to achieve automatic drainage and smart oil search; and using the characteristic of “dispersed oil aggregation” to achieve the purpose of oil droplet aggregation and realize the oil wall-type repulsion [72,73].

### 5.1. Nanoparticle Polymer Displacement Agents

Nanofluid is a special type of colloidal and non-Newtonian fluid prepared by adding different kinds of nanoparticles to different base fluids and mixing them in suspension [74]. In the field of enhanced oil and gas recovery, nanofluids are an emerging technology that has received extensive attention in recent years [75,76]. The advantages of nanotechnology in tertiary oil recovery can be attributed to the unique properties of nanomaterials. These include small nanoscale size, high surface-to-volume ratio, Pickering emulsion formation and stabilization, wettability alteration and interfacial tension reduction, and oil viscosity reduction ability. Accordingly, nanomaterials have a very promising future in EOR applications [77,78].

Kanj et al. [79] determined the available sizes of nanoparticles in reservoirs through nanofluid core drive experiments. The addition of nanoparticles to the fluid can significantly increase the recovery rate and improve the drilling results. This is achieved by changing the fluid properties, changing the wettability of the rock, reducing the drag in advance, enhancing the consolidation of sand grains, decreasing the interfacial tension, and increasing the mobility of the capillary captured oil. Cheraghian [52] conducted indoor experiments to determine the effect of nanoparticles on polymer-driven thick oil recovery. The results indicated that the introduction of nanoparticles resulted in a sharp increase in solution viscosity and a significant decrease in polymer adsorption rate. Compared with traditional polymer displacement agents, nanoparticle polymer displacement agents can realize the multiple effects of reducing polymer adsorption, increasing solution viscosity, improving crude oil recovery, and dispersing the breakthrough time of nanoparticles, all of which present significant advantages [80].

**Figure 13:**
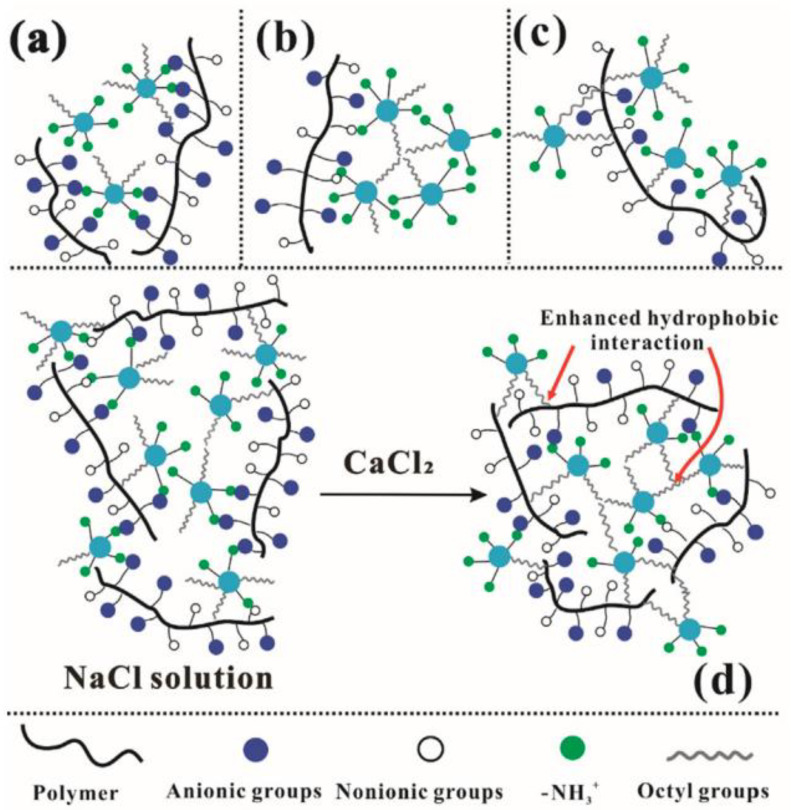
Amphiphilic nano-silica/HPAM complex preparation (**a**), Electrostatic attraction between OAS particles and polymer molecules. (**b**), Hydrophobic attraction between octyl groups. (**c**), Hydrophobic attraction between polymer backbones and octyl groups. (**d**), Hybrid network composed of OAS and polymer in a different salt solution [81].

**Figure 14:**
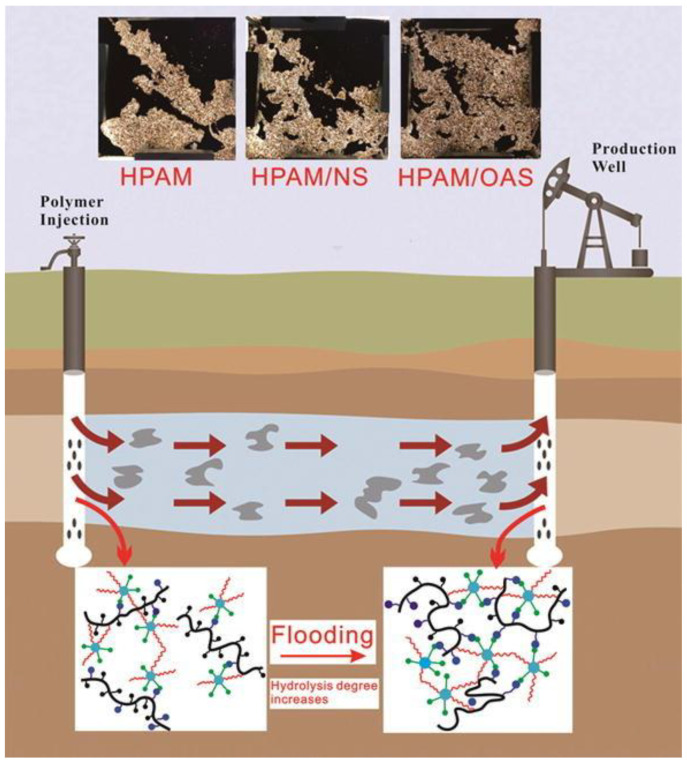
Amphiphilic nano-silica/HPAM complex oil repulsion process [81].

Cao et al. [81] prepared a composite oil displacement SiO_2_ NPs/HPAM containing nanoparticles through amphiphilic modification of SiO_2_ NPs and complexing with HPAM. The preparation of the SiO_2_ NPs/HPAM complex is displayed in Figure 13, and Figure 14 shows the oil displacement process, where OAS is the amphiphilic nano-silica. The amine-functionalized SiO_2_ NPs significantly improved the temperature and salt resistance of the HPAM. Compared with a non-particulate solution, the composite oil displacement system could increase the recovery by 12.3%, indicating that the incorporation of amphiphilic SiO_2_ NPs could solve the contradiction between viscosity enhancement and the injection of polyacrylamide-based polymers.

### 5.2. Nanoparticle Surfactant Displacement Agents

Low-surfactant oil drive is a crucial enhanced recovery method that can substantially increase recovery by reducing the oil–water interfacial tension and changing the pore wall wettability. However, the application of surfactants in high-temperature and high-salt reservoirs is challenging. Different from conventional surfactants, nanoparticles are distributed at the oil–water interface with a reduced thickness, and the electrostatic repulsion between particles makes the interfacial distribution more uniform, reducing the oil–water interfacial tension more significantly. As the nanoparticles can effectively reduce the critical micelle concentration, the solubilization ability of nanoparticles with the oil phase increases, and the crude oil in the reservoir forms a stable emulsion. This emulsification can improve the movability of the remaining oil. Moreover, the emulsion has a specific resistance coefficient that can improve the wave coefficient of the fluid [82,83].

**Figure 15:**
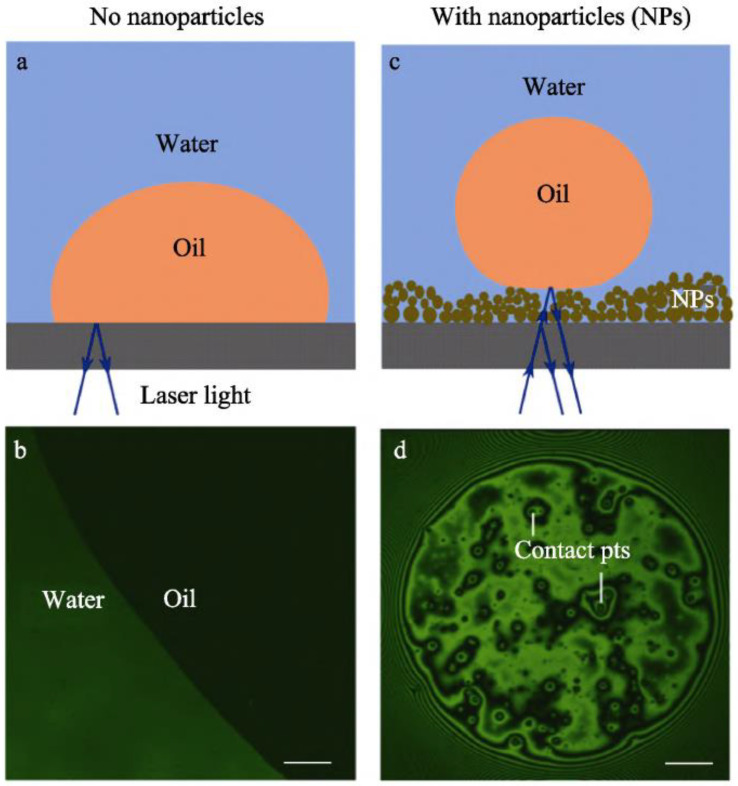
(**a**,**b**) Schematic and micrograph showing the oil droplet in contact with the glass substrate with no nanoparticles. The scale bar is 100µm. (**c**,**d**) The nanoparticles can stabilize a thin water film beneath the oil droplet and the droplet is in contact only with the topmost tips of the nanoparticle aggregates. The scale bar is 100 µm [84].

Li et al. [84] used several complementary techniques to visually and quantitatively analyze the alteration of the wettability of rock surfaces by gas phase SiO_2_ NPs. These techniques included confocal fluorescence, reflectance interference contrast microscopy (RICM), and droplet probe atomic force microscopy (AFM). The authors revealed that SiO_2_ NPs were adsorbed on glass surfaces to form porous layers with hierarchical microstructures and nanostructures. The porous layer traps a thin film of water, reducing the contact between the oil droplets and the solid substrate, the mechanism of which is displayed in Figure 15. The AFM measurements demonstrated that the addition of nanoparticles with a mass fraction of 0.1% reduced the adhesion of 20 μm-sized oil droplets by a factor of more than 400. In addition, the microfluidic experiments demonstrated that the change in wettability of SiO_2_ NPs could increase oil recovery by approximately 8%.

Mahmoudi et al. [85] observed a decrease in interfacial tension when SiO_2_ nanoparticles were used in combination with a surfactant. Moreover, this decrease exceeded the value when surfactant alone was used, indicating that the interfacial tension was reduced dramatically under the synergistic effect of nanoparticles and surfactant. The final recovery results revealed that when the surfactant concentration was 250 ppm, an increase in temperature and nanoparticle concentration significantly boosted recovery. In contrast, an increase in temperature and nanoparticle concentration had little impact on recovery when the surfactant concentration was 750 ppm. Silane-coated silica nanoparticles were added to conventional surfactants by Abhishek et al. [86] for use in carbonate reservoirs, and the resultant silane-coated silica nanofluids could influence the change in wettability to a more water-wet state. The optimal concentration for influencing the change in wettability was approximately 2 g/L, which brought it closer to the water-wet state. In addition, the oil displacement performance was significantly improved. Because of the change in wettability to a more water-wet condition, silane-coated silica nanoparticles could be employed to improve recovery in carbonate reservoirs, as the characteristic of nanoparticles is that they are widely used in the field of oil displacement. Table 4 introduces the application status of nanoparticles in oil displacement agents.

## 6. Functional Conformance Control and Water-Plugging Agents

High water content in oil wells is a common problem during oilfield development. Due to the non-homogeneity of the primary and secondary formations, the difference in fluid fluidity, and other reasons (such as operation failure and incorrect production measures), the dominant channel of injected fluid is formed in the formation. This results in the phenomena of coning-in, tampering, and finger-in. This makes the inefficiency or ineffective circulation of the injected fluid severe and can affect the economic and social benefits of the oilfield development. By injecting water plugging and regulating the agent system into the formation, the water absorption profile of the formation can be improved to realize the purpose of increasing oil and decreasing water. Therefore, control and water-plugging technology has proved an effective method for improving the development effect of oilfields and realizing stable production in oil reservoirs [87,88,89,90].

### 6.1. Smart Gel Agents

Smart gels are emerging materials that are structurally different from ordinary gels. The macromolecular side chains (or main chains) of smart gels contain polar, ionic dissociative, or hydrophobic groups. Once stimulated by the outside world, the conformation within the gel network will undergo corresponding changes and macroscopic changes in the morphology and performance of the whole gel will occur. Usually, these changes are reversible, meaning that once the stimulus disappears, the gel will return to the pre-stimulus state. Because of this special response mechanism, it is possible to design and control the microscopic molecular structure of the gel so that it can exhibit the smart characteristics expected by oilfield dissection [91,92].

The smart gel plugging agent’s plugging mechanism is based on the principle of the plugging agent in the reservoir forming a flexible viscoelastic gel, a plug of viscoelastic gel segments, or viscoelastic gel clusters as a whole of the viscoelastic gel. This renders it easier to squeeze the gel into the tiny pore spaces of the plugging layer, increasing the densification dramatically and blocking the pore flow channel of the stratum. In addition, the formation causes resistance to the flow, restricting or even preventing the passage of fluids following the injection of water steering to achieve the deeper part of the formation of the fluid flow steering to achieve the purpose of the sectional adjustment and increase the efficiency of water-driven flow.

**Figure 16:**
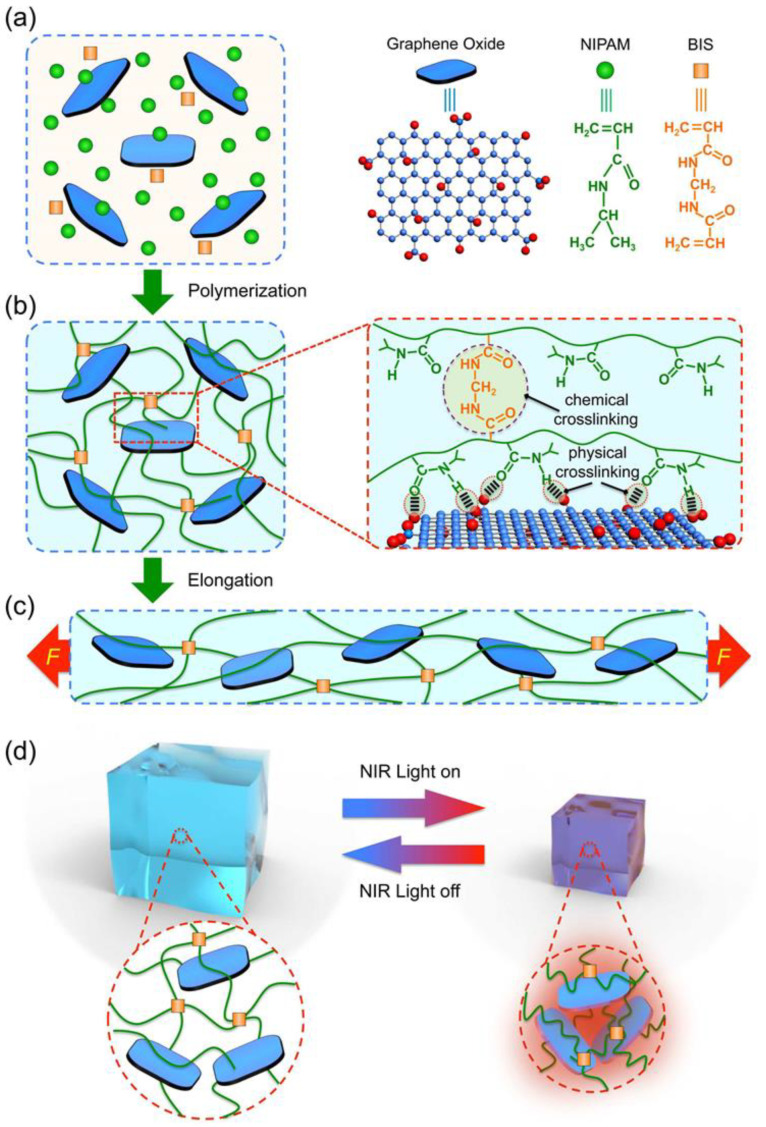
Schematic illustration of the fabrication process and performance mechanism of the proposed PNIPAM-GO nanocomposite hydrogels. (**a**) GO nanosheets are homogeneously dispersed in the monomer solution. (**b**) The PNIPAM-GO nanocomposite hydrogels are formed by both chemical and physical crosslinking, in which the PNIPAM chains are chemically crosslinked by BIS, and the hydrogen bond interactions between GO nanosheets and PNIPAM chains result in the physical crosslinking. (**c**,**d**) The PNIPAM-GO hydrogels exhibit ultrahigh tensibility (**c**) and reversible NIR light-responsive properties (**d**) [95].

When small chemical molecules (such as N and N-methylene bis-acrylamide) are used as crosslinking agents, an appropriate reduction in the crosslinking degree can effectively reduce the density of the crosslinking clusters in a gel network. Hence, clusters and clusters between the polymer chain will become longer, ensuring the entire polymer network is relatively uniform. When the gel is subjected to external stress, the constant length of the polymer chain can disperse the pressure and avoid any concentration of pressure in the short chain, improving the elasticity of the gel [93,94]. Shi et al. [95] proposed a novel near-infrared (NIR) light-responsive poly (N-isopropyl acrylamide)/graphene oxide (PNIPAM-GO) nanocomposite hydrogel with ultrahigh tensibility that was prepared by incorporating sparse chemical crosslinking of small molecules with physical crosslinking of graphene oxide (GO) nanosheets. The preparation process and performance mechanism are displayed in Figure 16. The PNIPAM-GO nanocomposite hydrogel was chemically crosslinked and physical crosslinking was formed, in which the PNIPAM chains were chemically crosslinked through BIS. The hydrogen bonding interactions between the GO nanosheets and PNIPAM chains resulted in physical crosslinking. The lower degree of chemical crosslinking significantly reduced the local crosslinking clusters of the smart gels and enhanced the degree of freedom of sliding of the polymer chains in the smart gels. When the degree of crosslinking was reduced to 0.01%, the smart gels could reach a strain of 3600% at fracture. The chemical crosslinking effect also ensured that the smart gel had a stable network structure.

### 6.2. Polymer Microsphere Agents

Polymer microsphere agents are a new type of water-plugging and regulating agent that has been developed in recent years. These plugging agents use hydrated and dissolved polymer microspheres on the pore throat of the formation channel sequentially to block the deep regulating technology. The driving mechanism of polymer microsphere agents is a crosslinking polymer microsphere with nanoscale initial particle size, and the diameter of the pore throat of the oil reservoir is designed to meet the requirements of “into the go.” After undergoing hydration and swelling, microspheres can attain the necessary particle size to block the pore throats, and the gel possesses adequate strength to fulfill the requirement of effectively blocking the formation of pore throats, redirecting the subsequent flow of fluids. Currently, research on the blocking mechanism of polymer microspheres is mainly focused on blocking the pore throat and matching the relationship between the particle size of polymer microspheres and the diameter of the core pore throat [78].

**Figure 17:**
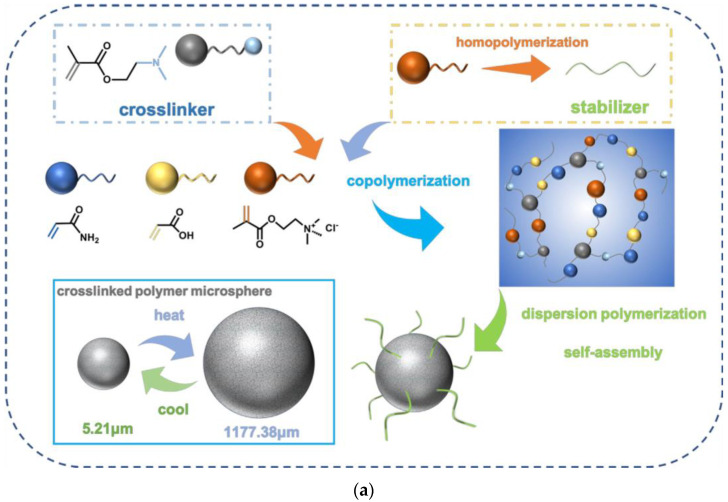

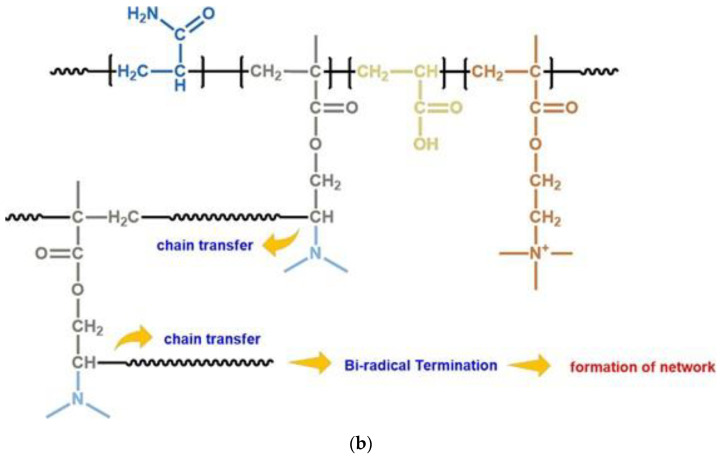
(**a**) Preparation process of crosslinked microspheres (c-PMAD). (**b**) The chemical formula of crosslinked polymer microspheres [98].

Polymer microspheres synthesized by dispersive polymerization have the advantages of low viscosity and good injectivity [96,97]. A temperature-responsive crosslinking polymer microsphere (c-PMAD) was prepared by Du et al. [98] using aqueous dispersion polymerization. c-PMAD was synthesized by this process and the chemical structure is displayed in Figure 17. Instead of conventional crosslinkers, 2-(dimethylamino) ethyl methacrylate (DMAEMA) was used in a chain transfer procedure to achieve crosslinking. In deionized water at 25 °C, it was discovered that the microspheres were spherical with a uniform surface and an average particle size of 5.21 m. Moreover, their thermal stability was relatively high. At a temperature of 66.5 °C, the microspheres exhibited upper critical solution temperature transition (UCST) behavior. When the temperature and the NaCl dosage increased, the size of the c-PMAD particles could decrease. Additionally, as the temperature reduced, the diameter of the c-PMAD shrank back to its original size, allowing it to be reused. In an 8 wt% NaCl solution, the UCST behavior of c-PMAD could be maintained.

Harsh formation environments can affect the driving performance of polymer microspheres. For example, if polymer microspheres can be synthesized that are responsive to the environment (such as temperature, pH, and shear), they can adopt the role of driving in complex formation environments, significantly improving the efficiency of the driving agent of the oil drive. Accordingly, research into smart polymer microsphere driving agents has become a trend in the development of driving materials [99]. By introducing a dynamic covalent network into a temperature-resistant gel, the double crosslinking gel PAM-FA-BGPP (PAFB) with temperature-responsive self-lubricating ability was constructed by Bai et al. [100]. Due to the thermal reversibility and dynamic equilibrium properties of the Diels–Alder reaction, PAFB can be decomposed at high temperatures of 80–140 °C to form a polymer aqueous lubricant with appropriate viscosity. This reduces its frictional resistance and effectively reduces the frictional resistance of the polymer microspheres to the microminiature pore throats during their deep migration in the formation, improving the deep mobilization ability of the polymer microspheres. The same research group introduced shear-responsive supramolecule FT (N-fluorenyl methoxycarbonyl-L-tryptophan) into chemically crosslinked polyacrylamide-sodium polyacrylate (PAM-PAANa) to synthesize a shear-responsive self-lubricating hydrogel (FT-PAM-PAANa). This effectively reduced the friction between the gel and the rock surface in the formation [101].

Water absorption and swelling are important indices for evaluating whether polymer microspheres can play the role of a regulator. Zhang et al. [102] prepared a smart polymer gel microsphere regulator (P(AA-AM C18DMAAC)@SiO_2_) with a multilayer assembly structure through multiple assemblies. This gel exhibited the property of slowly absorbing water at low temperatures and rapidly absorbing water at high temperatures. This characteristic is conducive to solving the contradiction between injectivity and plugging by absorbing water expanded in the depth of the formation and blocking the pore throat. Table 5 shows the statistics of the application status of conformance control and water-plugging agents and introduces the functional characteristics of intelligent gel agents and polymer microsphere agents in detail.

## 7. Prospectives of Functional Chemical Used in Oil and Gas Drilling Engineering

### 7.1. Drilling Fluid Loss Control

As a new research direction for drilling fluid treatment agents, smart plugging materials for drilling fluids have attracted the attention of many researchers due to their strong adaptability, outstanding plugging effect, and ability to cope with environmental changes. Moreover, smart gel plugging materials have the advantages of solid self-adaptation, they are unaffected by the size of fractures, the state of gel formation can be transformed due to changes in temperature, pH, and salinity, and the solidification time is controllable. Although this research direction is relatively novel and the plugging performance is outstanding, some problems remain that urgently need to be solved. (a) Similar to time-controlled gels, some of the gel plugging materials are not sufficiently temperature-resistant to be suitable for high-temperature strata. Accordingly, it is recommended that research on temperature resistance be strengthened in the future. (b) Shape memory plugging materials are characterized by high intelligence and outstanding plugging effects. However, the temperature-sensitive range of some shape memory materials is relatively narrow, the degree of temperature sensitivity is not high, the proportion of deformation is small, and the current research status is still in the experimental research and development stage. Moreover, the research data are relatively scarce and most products have yet to be used in field applications. (c) With the increasing depth of petroleum reservoir development, the problems of various complex loss formations have become increasingly apparent and smart lost circulation materials have become one of the essential methods for solving complex loss formations.

Research on new smart plugging materials is minimal and the direction is quite singular. It is suggested that the development of smart plugging materials should be strengthened in the future to create new products that could solve the problem of stratum loss at drilling sites and promote mutual exchanges between different disciplines.

### 7.2. Cementing

The study of the self-repair mechanism of microfractures in cementitious materials is a very new field and is of great significance for studying structurally and smartly integrated cementitious materials and improving the reliability and durability of cementitious materials at a lower cost. The self-repairing technology of cementing rings will become a new development direction of cementing technology that has significant theoretical research and engineering application value. Based on analyzing the existing research, we present the following suggestions for future studies. (a) In-depth exploration of the repair mechanism of cementing self-repairing cement paste is required to optimize the system. This would mainly include expanding the range of the intermixing ratio of composite self-repairing material cement paste, perfecting the performance of cement stone itself (such as pore structure), and establishing a complete evaluation system. The evaluation system would result in better and longer-lasting plugging and repairing and improved application prospects. (b) Exploration on the influence of temperature on self-repairing materials and self-repairing cement slurry should be conducted. This would solve the problem of cement slurry in different temperature intervals, especially the high-temperature performance of cement slurry. (c) We must gain a wider understanding of materials with self-repairing performance in various fields and carry out investigations on their influence on the performance of cement slurry and cement stone. Furthermore, we must analyze the self-repairing mechanism and search for new materials more suitable for the cementing field. (d) Current applications of self-repairing materials are mainly limited to indoor experimental research. Hence, they should be actively promoted in terms of applications and repeatedly verified, perfected, and improved through practice.

### 7.3. Hydraulic Fracturing

Currently, the main properties of fracturing fluids and materials are relatively homogeneous. Moreover, there is a requirement to develop smart, responsive fluids and materials for temperature, pressure, and other downhole environmental factors to meet the need to improve fracturing effects. For the sustainable exploitation of oil reservoirs, it is necessary to research clean fracturing fluids based on the current research and application of water-based fracturing fluids and the development requirements of hydraulic fracturing technology. Therefore, it is necessary to increase the efforts of experimental research further. For example, environmentally friendly and efficient VES fracturing fluids could be developed, and fracturing fluid systems should be optimized. Furthermore, studying the microstructure of VES fracturing fluids and the mechanism of the influence of reservoir conditions on the rheological properties would be important, as would expanding the scope of application of VES fracturing fluids, improving the effect of fracturing, and increasing production. Another aim is to make this technology one of the methods for the reforming of extra-low-permeability reservoirs and to explore using the new technology for reforming complex reservoirs.

Although there has been some research progress on nanomaterials in water-based fracturing fluids, many shortcomings still need to be addressed. Based on the analysis of existing research, the following suggestions are made for future studies. (a) Research on the application of nanomaterials in fracturing fluid breaking, formation particle transport control, rock surface wettability change, and proppant remains at the initial stages, and systematic and in-depth experimental research and mechanism analysis should be carried out in the future. (b) The agglomeration of nanoparticles has always been a critical issue that limits their applications. Agglomeration results in larger particle sizes, meaning nanoparticles lose their unique physicochemical properties due to their nanoscale size. Moreover, severe nanoparticle agglomeration can cause damage to the reservoir. To optimize the application of nanoparticles under formation conditions, the effects of different factors on the dispersion stability and the performance of the final fracturing fluid and the reservoir should be systematically investigated. (c) Currently, most of the applications of nanomaterials in water-based fracturing fluids are in the indoor research stage. Future research should incorporate field test results and use these as a criterion for continuous optimization and improvement of indoor research. This will promote the broad application of nanomaterials in water-based fracturing fluids more quickly.

### 7.4. Enhanced Oil Recovery

Smart nano oil displacement technology has become the focus of attention of the oil and gas industries due to its many advantages, including low price, no pollution, and strong environmental adaptability. Accordingly, it is expected to become a strategic replacement technology for improving recovery in the future. Smart nano oil displacement technology can realize crude oil recovery with high efficiency and low cost and potentially change the prospect of oil development. However, crucial technologies still need to be developed due to the limitation of related process technology conditions.

Although a preliminary understanding of the oil displacement mechanism of nanoparticles has been achieved, research on its applicability and surface modification remains at the system screening and effect evaluation levels. Restricted by weak knowledge of the theory of oil drive and recovery, the lack of cost reduction and efficiency mechanisms, and the ambiguity of safety and environmental protection risk evaluations, the pace of nano oil displacement technology needs to be revised. Currently, it is not easy to further promote the efficient and smooth implementation of the second development of the oilfield. It is suggested to take nanoparticles as the research basis, take chemical modification as the primary initiative, integrate fluid dynamics, statistical physics, materials science, and other functions, and endow nanoparticles with the target and intelligence to realize the multiple purposes and multiple uses of nano oil repellents. If “one agent with multiple functions” and “one agent with multiple uses” can be realized, this would bring new hope for developing future enhanced recovery technology.

### 7.5. Conformance Control and Water Plugging

In the field of smart gel plugging, single-responsive gels have been studied, while dual- or multiple-responsive gel systems with both temperature and pH have not been reported [103]. Preparing a smart gel system with dual-responsive temperature and pH with a synthesis route that has the advantages of mild preparation conditions, easy availability of raw materials, shorter synthesis routes, and higher yields would have great scientific significance and application value. Previous research has indicated that although the poly-N-isopropyl acrylamide system has better temperature responsiveness, its monomer has the disadvantages of teratogenicity and carcinogenicity, meaning it cannot satisfy the requirements of practical application in oilfields. Instead, non-toxic polyethylene glycol derivatives have been used to prepare gels with temperature sensitivity. In addition, polyamide dendrimers have strong pH-stimulated response properties, and exploring the use of polyethylene glycol-grafted polyamide dendrimers to prepare temperature- and pH-responsive gels represents an excellent research prospect [104].

With the continuous progress of nanomaterial research, they have been successfully applied in many fields by analyzing the existing water-plugging and regulating agent production needs. Currently, nanoparticle materials have four potential applications in terms of water-plugging and holding agents. (a) Nanoparticles are a good polymer base material enhancement agent that can improve the strength and temperature resistance of the original matrix material. (b) Nanoparticles have a large specific surface area and many surface reaction groups. These surface reaction groups can be adjusted by adjusting the rate of nanoparticles involved in the reaction to regulate the gel formation time. (c) Nanoparticles have a specific adsorption capacity in the gas/liquid and liquid/liquid interfaces and can be used to improve the stability of foams and emulsions through the addition of appropriate nanoparticles. (d) Achieving deep reservoir placement by leveraging the easy injectability and self-assembly characteristics of nanoparticles is another potential application.

Despite the achievements of smart anatomical agents in the field of petroleum development, gel microsphere anatomical agents developed and synthesized in the laboratory still exhibit some differences when applied in oilfields due to the different structures of the rock core. From the perspective of the actual effect of the oilfield, it is still necessary to carry out extensive exploration of composite microsphere anatomical agents.

## 8. Conclusions

With the expansion of oil and gas exploration and the development of deep, ultra-deep, unconventional, and other demanding oil and gas resources, the research and development of multifunctional materials and technologies will become the primary way to develop these oilfields efficiently. Functional gels and chemicals can adapt more successfully to various complex situations in oilfield development, significantly improve the plugging efficiency and recovery rate, and have a broad application prospect in oilfield drilling and extraction engineering. Researching the application methods of functional oilfield chemicals is not only important because functional oilfield chemicals are a crucial component of the petroleum industry’s development, but also because it simultaneously satisfies the economic and social benefits in this field by delivering practical usage results. The application of chemicals can also provide a more solid foundation for the development of oilfields and create conditions to improve the overall prosperity of the industry.

Frontier research in oilfield chemistry is an integral part of the development strategy of oilfield chemistry, and functional oilfield chemicals are the specific embodiment of the results of frontier research in oilfield chemistry, which is of great practical significance. The development of oilfield chemical materials is a product of the smart era, and the use of this high-tech knowledge in the field of oilfield drilling and extraction engineering will inject new content and vitality into the future development of oilfields. In addition, it will provide new opportunities to bring hope for future improvements to the level of oilfield development.

## Figures and Tables

**Table 1:** Statistics on the application status of functional drilling fluid chemicals.

Classification	Functional Materials	Function Characteristics	References
Drilling fluid flow regulator	Temperature-sensitive copolymer (PANA)	PANA can be a continuous network structure in solution and can change the rheological properties of fluid	(Xie et al., 2019) [27]
	Salt-responsive zwitterionic polymer (PAMN)	Salt-sensitive polymers can (to a certain extent) increase the ability of drilling fluids to re-sist salinization	(Sun et al., 2020) [29]
Filter loss reducer for drilling fluids	Terpolymer filter loss reducer	The filter loss reducer effectively maintained the rheological stability of the drilling fluid	(Thaemlitz et al., 2012) [30]
	A nano-lamellar silicate composite (AADS@LP)	The network strength of the polymer was improved, and the clay particles piled up tightly, forming an isolation membrane that reduced the filtration losses	(Shen et al., 2019) [31]
	A new generation of high-temperature-resistant filter loss reducers (Hostadrill 4706)	This filter loss reducer had excellent anti-salt properties, and it could significantly improve the rheological properties of drilling fluids	(Tomislav et al., 2003) [32]
Drilling fluid plugging materials	An aqueous dispersion of insoluble smart gels	The reaction of the smart gel with the OH- ions leached from the alkaline cement wall causes the gel viscosity to increase	(Ho et al., 2015) [40]
	Shape memory polymer (SMP)	Smart plugging working fluids based on SMP are automatically activated by temperature stimulation and produce deformation changes when they enter formations	(Zhao et al., 2015) (Lendlein and Kelch., 2010) [42,43]
	Lost circulation material (LCM)	The LCM is activated by the temperature of the bottom hole and can successfully seal the fracture’s mouth	(Mansour et al., 2017) [44]

**Table 2:** Statistics on the application status of functional cementing materials.

Classification	Functional Materials	Function Characteristics	References
Self-healing cementing material	The composite swelling mineral PC90M5B5	PC90M5B5 can improve the strength recovery rate, plugging efficiency, and self-healing effect of cement	(Qureshi et al., 2018) [8]
	A new additive oil swelling material (OSM)	Due to the oil-absorbing swelling of OSM, the self-healing cement paste expands and gradually occupies the space of the fracture	(Zhang et al., 2018) [50]
Nanoparticle cementing material	Nano-SiO_2_	Nano-SiO_2_ has a higher surface activity that significantly improves the stability of cement paste, including its compressive, splitting, tensile, and flexural strengths, as well as microstructural density, particularly enhancing the early strength of cement stone	(Sadrmomtazi et al., 2009) (Valipour et al., 2011) (Land and Stephan, 2012) [55,56,57]

**Table 3:** Statistics on the application status of functional hydraulic fracturing materials.

Classification	Functional Materials	Function Characteristics	References
Clean hydraulic fracturing fluids	A CO_2_-responsive surfactant isopropyl dimethylamine (EA)	EA was used as a thickener to develop a new viscoelastic surfactant-based fracturing fluid. The viscoelastic properties of this CO_2_-responsive VES fracturing fluid were excellent.	(Wu et al., 2018) [58]
	A supercritical CO_2_-clean foam fracturing fluid	This system can be extended to water-sensitive formations, preventing potential hydro-locking and preventing CO_2_ from affecting the internal structure of the micelles and altering the viscoelasticity of the fluids.	(Chen et al., 2005) [59]
Nanoscale hydraulic fracturing fluids	MgO and ZnO nanoparticles	The MgO and ZnO nanoparticles improved the thermal stability and the temperature resistance of the VES and exhibited higher viscosity yield.	(Gurluk et al., 2013) [63]
	Nano-SiO_2_	SiO_2_ nanoparticles increase the apparent viscosity and viscoelasticity of the solution, forming a three-dimensional network structure of the system.	(Fakoya and Shah, 2013) (Liu et al., 2020) [64,65]
High-temperature-resistant hydraulic fracturing fluids	A temperature-resistant thickener	This thickener significantly improves the viscosity and temperature resistance of the fracturing fluid, up to 232 °C.	(Funkhouser et al., 2010) [68]
	A new high-temperature fracturing fluid stabilizer (phenothiazine)	Phenothiazine was applied as a temperature stabilizer compounded with sodium thiosulfate in a specific polymer fracturing fluid system that could withstand formation temperatures of up to 260 °C.	(Carman and Gupta, 2014) [71]

**Table 4:** Statistics on the application status of functional oil displacement agents.

Classification	Functional Materials	Function Characteristics	References
Nanoparticle polymer displacement agents	A composite oil displacement SiO_2_ NP/HPAM containing nanoparticles	The amine-functionalized SiO_2_ NPs significantly improved the temperature and salt resistance of the HPAM. The incorporation of amphiphilic SiO_2_ NPs could solve the contradiction between viscosity enhancement and the injection of polyacrylamide-based polymers.	(Cao et al., 2019) [81]
Nanoparticle surfactant displacement agents	Nano-SiO_2_	Nanoparticles make the interfacial distribution more uniform, reducing the oil–water interfacial tension more significantly. The change in the wettability of SiO_2_ nanoparticles can increase the recovery rate by about 8%.	(Li et al., 2021) [84]
	Silane-coated silica nanoparticles	The silane-coated silica nanofluids could influence the change in wettability to a more water-wet state. In addition, the oil displacement performance was significantly improved.	(Abhishek et al., 2015) [86]

**Table 5:** Statistics on the application status of functional conformance control and water-plugging agents.

Classification	Functional Materials	Function Characteristics	References
Smart gel agents	A near-infrared (NIR) light-responsive poly(N-isopropyl acrylamide)/graphene oxide (PNIPAM-GO) nanocomposite hydrogel	The hydrogen bonding interactions between the GO nanosheets and PNIPAM chains enhanced the degree of freedom of sliding of the polymer chains in the smart gels. The chemical crosslinking effect also ensured that the smart gel had a stable network structure.	(Shi et al., 2015) [95]
Polymer microsphere agents	A temperature-responsive crosslinking polymer microsphere (c-PMAD)	Microspheres have high thermal stability. At 66.5 °C, the microspheres exhibit upper critical solution temperature transition (UCST) behavior. The particle size of c-PMAD decreases with the increase in temperature.	(Du et al., 2020) [98]
	The double crosslinking gel PAM-FA-BGPP (PAFB)	PAFB can be decomposed to form a polymer aqueous lubricant with appropriate viscosity. This reduces the frictional resistance of the polymer microspheres to the microminiature pore throats during their deep migration in the formation.	(Bai et al., 2020) [100]
	A smart polymer gel microsphere regulator (P(AA-AM C18DMAAC)@SiO_2_)	This gel exhibited the property of slowly absorbing water at low temperatures and rapidly absorbing water at high temperatures.	(Zhang et al., 2019) [102]

## Data Availability

Data sharing is not applicable to this article.
